# Impact of a digital platform on genetic counselling encounters in the screening context

**DOI:** 10.1038/s41431-026-02029-6

**Published:** 2026-02-13

**Authors:** Chloe Mighton, Alli Jan, Ling Lee, Sophie Bouffler, Lilian Downie, Marc Clausen, Clara Gaff, Yvonne Bombard, Zornitza Stark, Melissa Martyn

**Affiliations:** 1 Genomics Health Services Research Program, St. Michael’s Hospital, Unity Health Toronto, Toronto, ON Canada; 2 Institute of Health Policy, Management and Evaluation, University of Toronto, Toronto, ON Canada; 3 Murdoch Children’s Research Institute, Parkville, VIC Australia; 4 Australian Genomics, Parkville, VIC Australia; 5 Department of Paediatrics, Faculty of Medicine, Dentistry and Health Sciences, The University of Melbourne, Melbourne, VIC Australia; 6 Victorian Clinical Genetics Services, Murdoch Children’s Research Institute, Parkville, VIC Australia; 7 Walter and Eliza Hall Institute, Parkville, VIC Australia

**Keywords:** Genetic counselling, Genetics research

## Abstract

Digital tools for pre-test education provision and decision support could assist the scalability of opportunistic genomic screening. We evaluated the utility of a digital platform, the Genetics Adviser (GA), for supporting parental decisions about screening for additional findings in the paediatric acute care context. Parents of children who had completed ultrarapid diagnostic genomic testing in the acute setting were offered opportunistic screening following hospital discharge. Interested participants were provided with optional access to GA and offered pre-test genetic counselling (GC). GC sessions were audio-recorded, transcribed verbatim, and participant/counsellor interactions qualitatively analysed to examine the impact of GA use on counselling sessions. Surveys were administered: prior to and after pre-test GC; 1 month after return of results. One hundred and sixty-seven families were offered genomic screening and given access to GA. Family engagement with GA was 52% (87/167) overall, with three-quarters (81/119) of those who attended genetic counselling having engaged with GA. GA use impacted genetic counselling: in consultations where not all parents used GA, more concerns were raised and more questions asked about topics included in GA; GCs also spent more time clarifying values or understanding. GA users correctly answered more knowledge questions at every survey time point. Eighty-three per cent of post-result survey respondents believed GA contained enough information for them to make decisions about opportunistic screening without additional genetic counselling. These findings demonstrate the utility of GA in supporting the scalability of opportunistic genomic screening.

## Introduction

The use of genomic sequencing offers the opportunity to expand testing to include genomic screening, supporting early detection and intervention for genetic disease. However, there are challenges in scaling genetics service delivery to include screening. Traditional clinical genetic service delivery models incorporate pre- and post-test genetic counselling (GC), a clinical encounter with a specialised health care practitioner [1]. Pre-test GC includes elements of education and personalised decision support to help individuals make preference-sensitive decisions about undergoing genetic testing [2]. However, this traditional clinical genetics model is resource-intensive [3] and may not be appropriate or applicable for genomic screening settings. Novel approaches to health service delivery are needed to support broader availability of genomic screening.

Digital applications, including decision support tools, are one approach that could facilitate broader access to genomics. In clinical genetics, decision support tools have a long history of use in augmenting GC, particularly in prenatal and screening settings [4–6]. This is consistent with the purpose of decision support tools, which is to support preference-sensitive decision making, where the options do not have inherently superior health outcomes; the ‘right’ decision is that which aligns with an individual’s values. The more recent shift to digital tools has allowed for additional levels of complexity and customisation in information delivery and decision support [7, 8]. In genomic medicine, digital tools are used to support: risk assessment; pre-test education and counselling; return of results; and management [9]. A systematic review of patient-facing digital tools for delivering genetic services found positive effects on patient outcomes, including knowledge, psychosocial well-being, behaviour changes, family communication, and level of engagement [10]. Digital tools also led to efficiencies for health care practitioners [10].

Decisions about genomic sequencing can vary in their complexity: clinical testing may involve sequencing more than one person (trio testing) and, if screening is offered in addition to diagnostic testing, multiple decisions may be involved. Decisional multiplicity is known to complicate information provision and decision-making [11, 12]. Digital decision support tools are well-suited to help address this issue through their ability to provide information relevant to multiple decisions that families can work through at their own pace. For families in complex decision-making contexts, decision support tools are intended to augment, not replace, GC. Empirical evidence has demonstrated many benefits to digital tool use [10], but their impact on complex clinical consultations remains under-investigated.

To address this gap and generate insights to inform optimal use of digital decision support tools in genomic screening contexts, we evaluated the utility of the Genetics Adviser (GA) digital platform in supporting parents’ decisions about screening for additional findings in the paediatric acute care context. Additional findings were offered via a two-step model; participants were offered opportunistic genomic screening after results were returned from diagnostic genomic sequencing [13, 14]. We assessed uptake and use of the GA platform, and the impact of GA on the content of GC sessions.

## Methods

### Study design

The protocol for this study [14] provides details of the study design, including the modifications made to GA for this study. Uptake of screening results has previously been reported [13]. This study focuses on the impact of GA on GC and participant understanding of opportunistic genomic screening.

### Participants

Participants were parents of a child who had had ultra-rapid trio/duo diagnostic genomic testing in the acute care setting at one of 17 hospitals across Australia through the Acute Care Genomics study [15]. In this study, the diagnostic test was focused on the analysis of genes associated with the child’s condition. Parents were offered up to three genomic screening tests after return of diagnostic results: for the child (for treatable and non-treatable childhood onset conditions); for each parent (for adult-onset conditions with publicly funded management or treatment available); for couple carrier screening (1283 genes [16]— individual carrier status not determined). Screening was offered between September 2020 and May 2023.

### Genetic advisers use

The GA platform [17, 18] pre-test modules (in English) were modified to support parental education, decision-making and consent about genomic screening for this study. Parents were provided access to GA prior to pre-test counselling; participants were not required to use it. GA was accessed via a web interface on the participant’s own device. Unique usernames were provided to each parent to allow tracking of individual users. Data was collected on login and duration of use of GA (captured for the most recent login). As the aim of our study was to explore the impact of GA use on decision-making about, and GC for, genomic screening, we determined a threshold for meaningful engagement with DA. We defined ‘use’ of GA as logging in and spending at least 5 min using the platform. We determined 5 min as sufficient time to orient to the platform and engage in an area of interest. Use of GA was defined at the individual level (did/did not use GA) and at the family level (concordant, discordant and no use of GA).

### Counselling data collection and analysis

The focus for counselling data collection and analysis was the family, including family use of GA. All parents attended pre-test counselling with a health professional trained in GC for genomic screening, regardless of whether they had used GA or not. Counselling sessions were audio-recorded with consent, transcribed verbatim by a professional transcription service and analysed. Qualitative data were managed using NVivo 14. Data were structurally coded based on the speaker and content or prompting of their speech using a modification of a previously published method [19, 20] (Supplementary Fig. 1). GC information provision was coded according to whether information was ‘offered’ by the GC or ‘prompted’ by the participant; GC questions clarifying values or assessing understanding were coded separately. Participant speech was coded for three types of behaviour: ‘assertions’ of feelings or beliefs; ‘concerns’ regarding participation; and ‘questions’ about screening. ‘Questions’ were subdivided into categories based on whether the information had been ‘addressed’ in GA, if it was ‘novel’, or if it was based on a ‘misconception’. All information not directly related to genomic screening was coded as ‘other’. Time and occurrences are reported.

Coding was conducted by one independent reviewer (AJ) who did not participate in GC, with 10% of transcripts recoded by a second reviewer (CM) who was not informed of the purpose of the research or the different categories of participants prior. Inter-coder reliability was calculated with a Cohen's Kappa score. The primary reviewer was blinded as best as possible to GA use when coding; however, many participants referred to the use of GA within the transcript.

Verbal behaviours were compared based on participant use of GA with the analysis focusing on three groups of participants: concordant (all parent participants in the genetic counselling session used GA); discordant (one participant used and one did not use GA); and no use of GA. GC appointments with only one participant were categorised based on that person’s GA use and not couple use. Bivariate analyses of coded verbal behaviours were examined with two-sided t-tests, with equal or unequal variances as appropriate.

Separately, appointment transcripts were analysed using inductive content analysis [21]. One reviewer (AJ) first read through each transcript and selected data segments pertaining to the perception of GA or its influence on participation in genomic screening. These chunks of data were then coded by AJ for big picture categories, which were verified in discussion with a second member of the research team who did not participate in counselling (MM).

### Participant survey data collection and analysis

The focus of analysis for participant surveys was the individual parent—their GA use and survey completion. Participants were asked to complete optional surveys after use of GA (where used) but prior to counselling (S1), after counselling (S2), and about one month after return of results (S3). Details of the content of surveys [14] and findings [13] have been published previously. The number of knowledge questions answered correctly for each respondent at each time point was determined by collapsing responses into correct/incorrect answers. Group means and standard deviations were used to assess knowledge between groups (individual GA users/non-users) and across time. The pre-counselling and post-counselling surveys asked seven True/False/Unsure knowledge statements and two multiple-choice knowledge questions. The post-result survey contained three True/False/Unsure questions; the number of knowledge questions correct post-results was multiplied by three to allow comparison across surveys. Validated measures included in the surveys (DCS [22], STAI [23, 24], DR [25], GOS [26], FACToR [27], Satisfaction with genetic counselling [28], TMSI [29]) were calculated in line with the authors’ instructions. DR is reported as per a previously published categorisation system [30]. Demographic data were used for univariate associations of GA uptake and knowledge, analysed with Pearson chi-squared tests, t-tests, or Wilcoxon rank-sum tests analysis as appropriate to the type of data. Quantitative data were analysed in Stata 18.0 [31] and R [32]. A *p*-value of less than 0.05 was considered statistically significant. Open-ended survey responses were inductively coded by one reviewer (AJ) and verified by a second member of the research team (MM).

### Ethical approval

Ethical approval for this research was obtained from the main HREC, the Melbourne Health Human Research Ethics Committee, as part of the Australian Genomics Health Alliance protocol (HREC/16/MH/251). Informed consent to participate in this sub-study was obtained from all participants as required by the HREC.

## Results

**Fig. 1:**
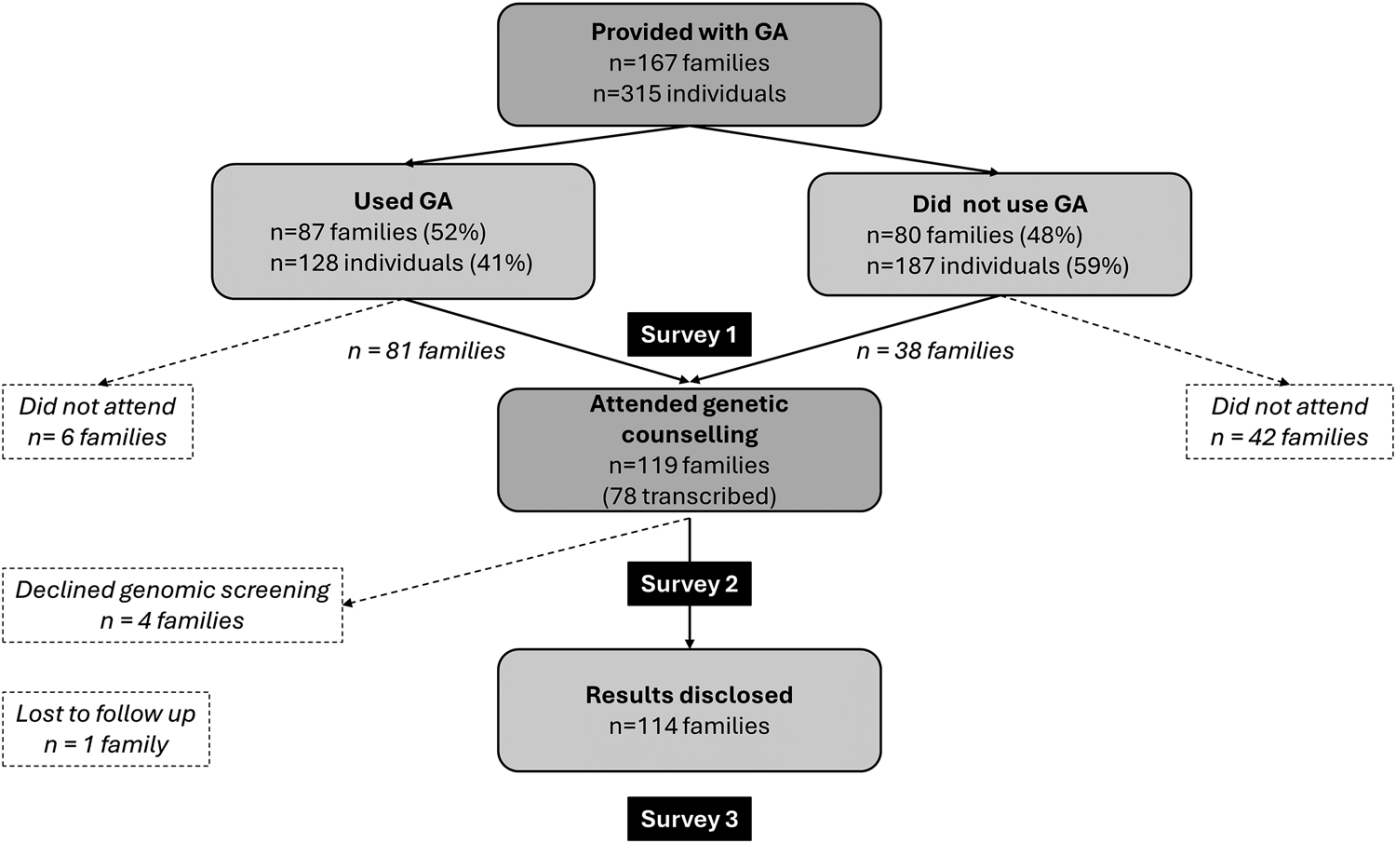
Overview of study participation. Study interventions were access to GA and genetic counselling (shown in dark grey boxes).

One hundred and sixty-seven families who were offered genomic screening were given access to a modified version of the GA patient platform [17, 18] (Fig. 1).

### Engagement with GA

GA was used by 74 mothers and 54 fathers from 87 families, giving a family engagement of 52% (87/167) and individual engagement of 128/315 (41%). No participant characteristics (e.g. demographic and family features) were associated with engagement with GA (Supplementary Table 1*)*. GA utilisation metrics showed individuals spent a median of 12 min (IQR 7 min to 19 min) using GA at their most recent login.

### Perspectives of GA

Family and individual survey response rates have been published elsewhere [13]. To recap, response rates by individuals were: pre-counselling survey (S1) 58/209 (28%); post-counselling survey (S2) 98/223 (44%); post-result survey (S3) 50/209 (24%). The pre-counselling survey (S1) was returned a median of 1 day after it was sent out (IQR 0–3). Median time between pre-test counselling and return of the post-counselling survey (S2) was 16 days (IQR 4–29); median time between return of results and return of post-result survey (S3) was 26 days (IQR 8–55). Median time between post-test counselling and return of results surveys (S2 and S3) was 149 days (114–215 days).

Most GA users who completed the pre-counselling survey indicated they used GA for between 5–20 min (30/45, 67%). The majority of survey respondents who used GA rated the amount of information in GA as ‘about right’ (42/45, 93%). The videos and family example scenarios were identified as the most useful aspects of GA from responses to an open survey question. Respondents appreciated the information included, as well as the layout and structure; ‘*it broke things down into easy-to-read language and helped with processing the steps to decision making’ (Family 23)*. Inductive content analysis of the genetic counselling transcripts supported the survey findings, with comments about the value of the case studies and that GA was ‘straightforward’ to use. One father did report that he had found it overwhelming and had relied on his wife to use it. Two other couples also reported that one member of the couple had reviewed on behalf of both of them. This analysis also identified three participants who specifically stated that GA had influenced their decision about participating in genomic screening: ‘*I initially didn’t want to find out at all and then after actually watching the videos and doing the [GA] you sent through… [I] changed my mind on it’(Family 11)*.

The post-counselling survey asked GA users if they believed GA contained enough information for them to make decisions about each of the screening tests without the additional counselling provided: 83% (29/35) of respondents agreed it did. Six participants indicated they still required GC input after GA use to decide on genomic screening; 4 provided reasons included outstanding questions (*n* = 2), and complex medical situations (*n* = 2). Of note, among survey respondents, the only factor associated with increased use of GA (Supplementary Table 2) was a high score for information seeking in the validated measure *Threatening Medical Situation Inventory* *[*29], which assesses the tendency to seek or avoid information under stress. Decisional conflict [22] and State Trait Anxiety [23, 24] were not associated with GA use.

### Analysis of genetic counselling sessions

One hundred and nineteen families of the 167 provided access to GA subsequently attended genetic counselling: 81 of these had at least one parent who had used GA and 38 families had no parent use GA. Six families that used GA did not go on to attend genetic counselling.

When comparing family genetic counselling appointment attendance by GA use for all 119 families, we noted only one family of 44 with concordant GA use required a repeat appointment (2%), compared to families with discordant or no GA use (11/75, 15%, *χ*^2^ = 4.698, *p* = 0.030).

Genetic counselling sessions were recorded for 78 families (*n* = 78/119, 66%). In two families, parents attended counselling separately, giving a total of 80 counselling transcripts. Sixteen genetic counsellors and one clinical geneticist were involved in appointments. The codebook used to analyse transcripts identified verbal behaviours by speaker (Table 1). Inter-coder comparison resulted in a weighted Cohen-Kappa score of 0.90. For transcript analysis, GA use was classified as ‘concordant’ (all parents used GA; *n* = 29), ‘discordant’ (one parent used and one did not; *n* = 22), and ‘not used’ (*n* = 29). Impact of GA use on counselling was compared for concordant v discordant/not used families, as clinically any effect of GA use on counselling would only be apparent with concordant use; with discordant use, GCs would still have a ‘non user’ in the consultation and so have to counsel accordingly.

**Table 1:** Codebook of qualitative codes.

Code	Description	Examples
Speech related to screening		
Genetic counsellor speech		
Offered information	Information delivered that was freely offered by the GC	‘*So I thought today we might start off by talking about reproductive carrier screening…’(Family 7)*
Prompted information	Information delivered by the GC in response to a statement or question by the participant	‘*To directly answer your question, yes, it [couple carrier screening] would only tell you about the things you’re both a carrier of’(Family 10)*
Clarifying values/understanding	Questions eliciting personal values or understanding of content	‘*How do you think you would feel if you found out about a condition that there wasn’t any treatment available for?’(Family 24)*
		‘*Maybe we can start with your understanding of… what additional [screening] you can have?’(Family 25)*
**Participant speech**		
Assertions	Statements of rationale for participation; includes affirmative and negative statements and opinions	‘*Having such a complex child, it’s something we need to do because if there’s another puzzle piece it’s important that we know about it’ (Family 26)*
		‘*If you can’t do anything about it, I know about it so I can make sense of it’(Family 27)*
Concerns	Statements about anticipated problems, including worries, fear and frustrations	‘*The childhood-onset is a little bit scary because that one’s not completely preventable stuff’(Family 26)*
		‘*I’m definitely going to be way overthinking every little thing’(Family 28)*
Questions	Statements or explicit questions that result in prompted information	
- Novel	A question about participation or screening that has not been explained in GA	‘*Will this allow us to have a bit more understanding of our genomes to plan on IVF?’(Family 29)*
- Addressed	A question addressed and answered in GA	‘*Is there a possibility they find like a condition that’s never existed before?’ (Family 30)*
- Misconception	A question based on a poor understanding of the concepts being discussed	‘*When you do a DNA check, you can figure out how long a person is going to live…can we do that too while we're at it?* ’*(Family 12)*
**Other speech**		
Other	Anything in the appointment that is not related to the discussion of screening	Preamble, family tree documentation, discussion of primary diagnosis, child’s health, etc.

### Impact of GA use on genetic counselling

Participants were scheduled pre-test counselling appointments of standard length for the service at which they were seen. Table 2 shows the content of counselling sessions, comparing those where all parents had used GA, with non-users and discordant users. Looking at speech related to screening, 20% less time was spent discussing screening in families with concordant GA use compared to discordant and no use (*p* = 0.037). Counsellors spent more time clarifying values or understanding in appointments with families that had not (both) used GA (*p* = 0.037). Participants who had not all used GA raised more concerns during their appointments (*p* = 0.024) and asked more ‘addressed’ (*p* = 0.038) questions than families with concordant GA use. Counsellors also spent more time providing ‘prompted’ information to those who had not (both) used GA (*p* = 0.003).

**Table 2:** Time and occurrence of verbal behaviours in counselling transcripts, compared by use of GA.

	All counselling transcripts	All parents used GA	Not all parents used GA	Significance
	(*n* = 80)	(*n* = 29)	(*n* = 51)	
**Time (minutes), mean (SD)**				
Total	40.41 (15.69)	39.51 (18.94)	40.92 (13.68)	*t* = 0.354
				*p* = 0.725
Total spent discussing screening	22.81 (9.97)	19.74 (10.74)	24.56 (9.16)	*t* = **2.122**
(total minus ‘other’, as defined in Table 1)				*p* = **0.037**
Information offered (GC)	10.54 (4.57)	10.09 (5.32)	10.80 (4.11)	*t* = 0.665
				*p* = 0.508
Prompted information (GC)	3.75 (4.36)	2.08 (2.99)	4.70 (4.74)	*t* = **3.027**
				*p* = **0.003**
Clarifying values/understanding (GC)	0.82 (0.59)	0.64 (0.48)	0.93 (0.63)	*t* = **2.119**
				*p* = **0.037**
Assertions (pt)	2.61 (2.76)	2.96 (3.09)	2.41 (2.57)	*t* = -0.851
				*p* = 0.397
Concerns (pt)	0.25 (0.47)	0.12 (0.29)	0.32 (0.53)	*t* = **2.194**
				*p* = **0.031**
**Occurrences (number of times), mean (SD)**				
Clarifying questions (GC)	5.16 (3.34)	4.28 (3.30)	5.67 (3.28)	*t* = 1.817
				*p* = 0.073
Concerns (pt)	0.96 (1.49)	0.52 (1.06)	1.22 (1.64)	*t* = **2.312**
				*p* = **0.024**
Total questions (pt)	5.84 (6.31)	4.28 (5.03)	6.73 (6.81)	*t* = 1.834
				*p* = 0.071
‘Novel’ questions	2.13 (2.81)	1.66 (2.72)	2.40 (2.85)	*t* = 1.146
				*p* = 0.256
‘Misconception’ questions	0.26 (0.57)	0.24 (0.58)	0.27 (0.57)	*t* = 0.248
				*p* = 0.805
‘Addressed’ questions	3.13 (2.35)	2.14 (2.76)	3.69 (3.73)	*t* = **2.118**
				*p* = **0.038**

GC refers to counsellor codes and pt to patient codes.

### Knowledge and decisional conflict

The use of GA significantly impacted individual participant knowledge, with GA users answering more knowledge questions correctly at every time point (Table 3a*)*. Genetic counselling did not impact knowledge (Table 3b). Other factors that were positively associated with higher post-counselling knowledge scores were female gender (*p* = 0.004) and younger age (*p* = 0.002). Following counselling, decisional conflict [22] did not differ between GA users and non-users (*χ*^2^ = 3.603, *p* = 0.165) (Supplementary Table 2).

**Table 3:** Impact of GA use [a] and counselling [b] on the number of knowledge questions answered correctly across survey timepoints.

3a Effect of GA use on knowledge at each survey time-point			
	Used GA	Did not use GA	Significance (p)
	Mean (SD)	Mean (SD)	
Pre-counselling survey (S1) (*n* = 34, 10)	6.4 (1.7)	4.2 (2.1)	*p* = **0.001**, *t* = –**3.541**
Post-counselling survey (S2) (*n* = 63, 27)	6.2 (1.8)	4.9 (1.7)	*p* = **0.003**, *t* = –**3.081**
Post-result survey (S3) (*n* = 41, 9)	2.4 (0.8)	1.8 (0.8)	*p* = **0.032**, *t* = –**2.212**

3b Effects of counselling on knowledge pre- and post-counselling			
	Pre-counselling (S1)	Post-counselling (S2)	Significance (p)
	Mean (SD)	Mean (SD)	
All respondents	5.6 (2.4)	5.8 (1.8)	*p* = 0.530, *t* = –0.632
(*n* = 47, 90)^a^			
Used GA	5.9 (2.4)	6.2 (1.8)	*p* = 0.561, *t* = –0.586
(*n* = 37, 63)^a^			
Did not use GA	4.2 (2.1)	4.9 (1.7)	*p* = 0.346, *t* = –0.981
(*n* = 10, 27)^a^			
*Knowledge questions at S1/2 are:*			
**If I choose to receive** **adult-onset additional findings, doctors will look at:**			
Genes for all known genetic conditions.			
Genes for a select group of adult-onset genetic conditions that have a known treatment or intervention.			
Genes for a select group of adult-onset genetic conditions for which no treatment or intervention is available			
Genes related to my child’s health condition			
**If I choose to receive** **childhood-onset additional findings for my child, doctors will look at:**			
Genes for all known genetic conditions that affect adults and children			
Genes for a select group of genetic conditions that affect children have known treatment or intervention.			
Genes for a select group of genetic conditions that affect children for which treatment or intervention may or may not be available			
Genes related to my child’s health condition			
**True/False/Unsure questions**			
No additional finding in myself completely rules out all other genetic conditions for myself			
No additional finding in myself completely rules out all other genetic conditions for my family			
An additional finding in myself could be relevant to other family members			
An additional finding in my child means they will definitely get the condition			
An additional finding in my child may reveal a condition with no known treatment			
If we choose to have genetic carrier screening, we will each receive our own individual results			
No additional findings on the couple’s carrier screening means we still have a low chance that a future child may have a different genetic condition			
*Knowledge questions at S3 (all True/False/Unsure) are:*			
No additional finding in myself completely rules out all other genetic conditions for myself			
No additional finding in myself completely rules out all other genetic conditions for my family			
The results a couple receive from the genetic carrier screening analysis cannot be used with a new partner			

Impacts assessed using t-tests. Mean number of knowledge questions answered correctly given for grouped individuals. Number of respondents displayed as (*n* = *n* of column 2, *n* of column 3).

^a^Denotes a group with both paired and unpaired data; these data were analysed using partially overlapping t-tests.

## Discussion

As applications of genomic screening grow, alternative service delivery models are required to prevent resource pressures from becoming a limiting factor in offering screening. In a clinical setting, augmenting pre-test genetic counselling with digital tools is a potential solution; however, the impact of decision tools on complex clinical consultations is under-explored in the genomic screening setting. This study reports results of an approach focused on clinician-patient communication to examine the impact of a decision support tool in active decision-making related to opportunistic genomic screening during genetic counselling appointments. Our results show use of the web-based digital platform GA improved patient understanding of genomic screening and impacted subsequent genetic counselling, reducing the amount of consult time spent discussing genomic screening and the number of families requesting more than one pre-test genetic counselling session. These findings demonstrate the utility of GA in supporting education and decision support for genomic screening and suggest that such digital tools have the potential to help scale genomic screening.

One of the goals of digital tool use is to support clinical service efficiencies. We noted efficiencies in the number of pre-test counselling appointments, with only one family that used GA requiring more than one pre-test counselling appointment. We also noted less time spent discussing screening in consultations with GA users. Our study design does not enable us to draw causal conclusions between GA use and these findings, yet we did not find any differences in participant characteristics associated with GA use. In terms of time efficiencies, previous studies designed to compare consultation time found reductions in consultation time among digital tool users [10, 33]. In our study, families in complex medical situations who had already completed genomic testing were scheduled for a standard pre-test counselling session to discuss genomic screening. Many families were seen by the genetic counsellor who had provided diagnostic test counselling. Overall consult time did not differ based on GA use. We noted that about half the consultation time was spent on matters other than screening, which may suggest families using the time to meet unmet needs, and/or counsellors taking the opportunity to provide other support pertinent to the family. In this complex cohort already familiar with genomic testing, counselling for opportunistic screening required only 20–25 min (on average) of the scheduled consultation time. Although evaluation in other cohorts, including those less familiar with genomic testing, is needed, this result suggests that focused genetic counselling about opportunistic genomic screening requires less time than pre-test genetic counselling and thus could inform future service design.

Our study explored decision tool use to augment genetic counselling—it was not intended to replace it. Some studies have suggested that decision tools may replace genetic counselling, but caution may be needed in complex clinical populations. In particular, care must be taken not to exacerbate existing inequalities. Whilst digital solutions offer potential for optimising the use of healthcare professionals [34], and improve patient-centred care for families [17], they also have the potential to widen inequality in disadvantaged populations [35]. Service designs must continue to co-design and evaluate for all, including those with low digital literacy or poor access to digital infrastructure [36]. One benefit of digital tools is to support education and decision support, which allows genetic counselling time to be spent on more nuanced, patient-centred discussions of values and decision-making factors. Although some of our participants reported that they felt equipped to make their decision about genomic screening without genetic counsellor input, others valued the genetic counselling support. Allowing participants to opt out of pre-test counselling may be feasible when participant needs are met by the digital platform, reserving genetic counselling resources for patients who are most in need. A challenge will be in determining which patients require genetic counselling and ensuring those who opt out of genetic counselling are sufficiently informed and supported.

Genomic screening applications are increasingly being trialled and implemented, including opportunistic screening for additional findings, couples-based carrier screening [16], population-based screening for actionable conditions [37] and genomic newborn screening [38, 39]. Workforce capacity has been identified as a key issue facing national genomics initiatives [40]. It must be recognised that genomic screening creates new needs for clinical follow-up—all those with positive genomic screening findings require clinical follow-up to identify any associated symptoms. Quantification of the impact of this will be important to support decision-making in publicly funded healthcare systems about the introduction of screening initiatives. Alongside this, new paradigms for patient education and decision support are needed. Integrating a digital tool to supplement GC time can help to optimise the use of the genetic counselling workforce and is likely to benefit the scalability of genomic screening. Indeed, ongoing population genomic screening initiatives have employed digital enrolment and consent [37]. Given the complexity of our cohort, as well as the two-step model - meaning that patients had already received clinical support related to their decision to have genomic testing for the primary diagnostic indication - evaluations are needed in other cohorts, such as population screening, to examine how genetic counselling practice would be impacted by using digital tools in this setting. Additionally, while not explored in our current study, digital platforms can also support the return of results [10], further promoting the feasibility of population genomic screening.

Our study findings must be considered in light of several limitations. Whilst GA is now available in multiple languages, at the time of this study, GA was only available in English, which limited its accessibility. At the time of the study, the platform also only retained login data for the most recent login, so we did not have time data for earlier logins for individuals who accessed GA multiple times. However, most participants self-reported using GA for 5–20 min, which is consistent with the data from the platform. We did not collect data on reasons for declining to use GA. Digital tools can also be used to support the return of results; however, this was not tested in the current study and is an area that warrants future research. Our genetic counselling transcript analysis was designed to classify genetic counsellor and parent participation in counselling about genomic screening; we did not make any attempt to analyse the quality of those interactions. As such, we can make no comment on the value of ‘other’ conversation in the consultation. Our initial review of transcripts did not reveal obvious differences between GA users and non-users in counselling topics discussed, but we note our content analysis was not designed to explore detailed differences in consultations between GA users and non-users. We also note that counsellors and families were aware that sessions were being recorded and this may have impacted the consultation. Finally, our population was medically complex, and findings cannot necessarily be extrapolated to the general population.

As opportunistic genomic screening is increasingly offered in clinical populations, approaches are needed that allow clinicians to focus on decisions around diagnostic testing, while providing alternative supports for screening decisions. One approach is the augmentation of genetic counselling with digital tools. We found that digital tools can effectively support information delivery; transferring standardised information provision to the tool can allow genetic counsellors to allocate time to providing more individualised care tailored to each patient’s needs, which will help to promote value-congruent decisions. These findings demonstrate the utility of GA in supporting opportunistic genomic screening and suggest that digital tools have the potential to support the scalability of genomic screening.

## Supplementary information

Supplementary Information

## Data Availability

The data that support the findings of this study are available on request from the corresponding author (MM). The data are not publicly available due to containing information that could compromise research participant privacy.
