# Supplementary material for: Impact of a digital platform on genetic counselling encounters in the screening context
Source: Eur J Hum Genet. 2026 Feb 13;34(5):715–22. doi: 10.1038/s41431-026-02029-6 (PMC13172509; doi:10.1038/s41431-026-02029-6)
Supplement: Supplementary file 1 — Supplementary Information [file 41431_2026_2029_MOESM1_ESM.docx]

**Supplementary Information**

Supplementary Figure 1 – Flowchart showing deductive coding used for analysis of Genetic Counselling session transcripts


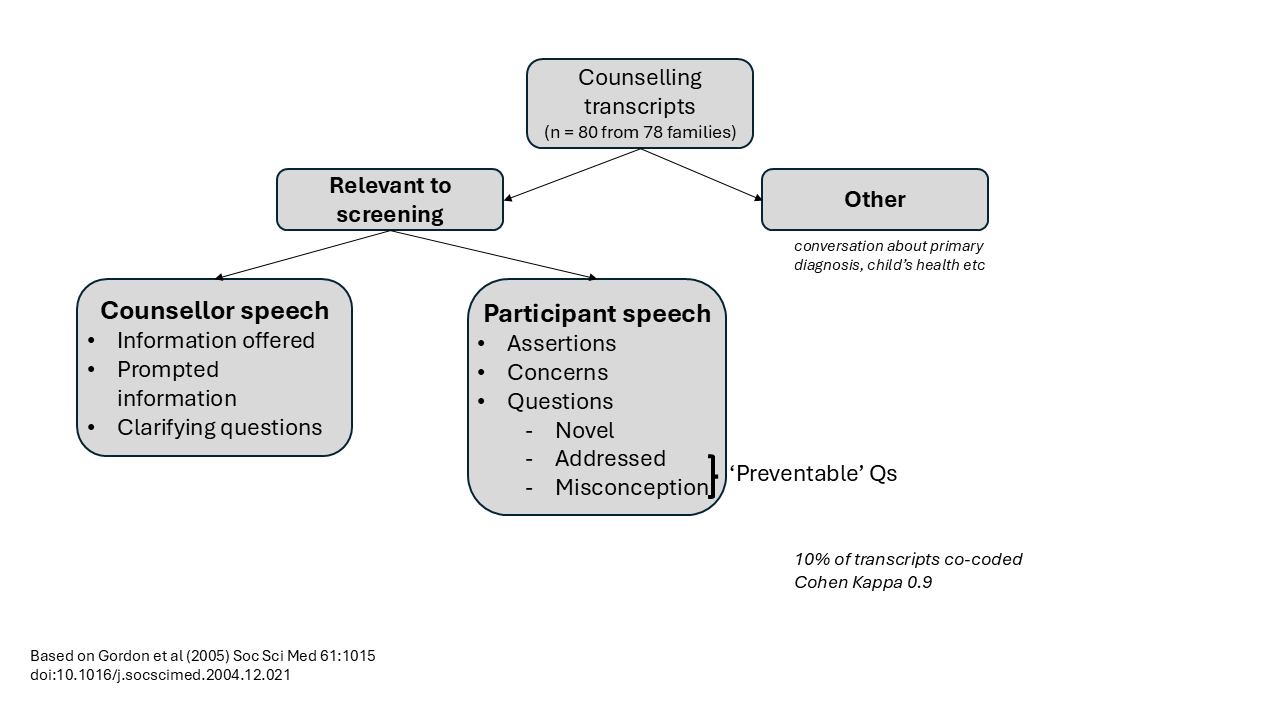


# **Supplementary Table 1 – Demographic factors of participants who used and did not use GA**

| **Category** | **Participants (n=315)** | **Used GA**  **(n=128)** | **Did not use GA**  **(n=187)** | **Significance** |
| --- | --- | --- | --- | --- |
| Age [mean (SD)] | 34.50 (6.55) | 34.88 (5.61) | 34.25 (7.13) | p=0.3846  t=-0.8707 |
| Parent role, n (%) | | | | |
| Mother | 165 (52.4%) | 74 (57.8%) | 91 (48.7%) | p=0.110  *χ*^2^=2.550 |
| Father | 150 (47.6%) | 54 (42.2%) | 96 (51.3%) |  |
| Ethnicity | | | | |
| Aboriginal | 8 (2.6%) | 2 (1.7%) | 6 (3.3%) | p=0.187  *χ*^2^=11.27 |
| African | 6 (2.0%) | 2 (1.7%) | 4 (2.2%) |  |
| Asian | 85 (28.1%) | 31 (25.6%) | 54 (29.7%) |  |
| European | 207 (68.3%) | 87 (71.9%) | 120 (65.9%) |  |
| Hispanic | 8 (2.6%) | 4 (3.3%) | 4 (2.2%) |  |
| Middle Eastern | 27 (8.9%) | 10 (8.2%) | 17 (9.3%) |  |
| Oceanian | 15 (5.0%) | 3 (2.5%) | 12 (6.6%) |  |
| Other | 10 (3.3%) | 3 (2.5%) | 7 (3.9%) |  |
| Previous diagnosis | | | | |
| Yes | 163 (51.8%) | 65 (50.8%) | 98 (52.4%) | p=0.777  *χ*^2^=0.080 |
| No | 152 (48.3%) | 63 (49.2%) | 89 (47.6%) |  |
| Deceased child | | | | |
| Yes | 70 (22.2%) | 34 (26.6%) | 36 (19.3%) | p=0.125  *χ*^2^=2.350 |
| No | 245 (77.8%) | 94 (73.4%) | 151 (80.8%) |  |
| Main language used | | | | |
| English | 127 (87.6%) | 78 (89.7%) | 49 (84.5%) | p=0.355  *χ*^2^=0.856 |
| Other | 18 (12.4%) | 9 (10.3%) | 9 (15.5%) |  |
| Highest level of education | | | | |
| Less than secondary | 13 (9.0%) | 9 (10.2%) | 4 (7.1%) | p=0.266  *χ*^2^=3.956 |
| Secondary | 43 (29.9%) | 21 (23.9%) | 22 (39.3%) |  |
| Post secondary | 49 (34.0%) | 32 (36.4%) | 17 (30.4%) |  |
| Graduate and beyond | 39 (27.1%) | 26 (29.6%) | 13 (23.2%) |  |
| Self-reported annual combined household income (AUD) | | | | |
| <$39 999 | 9 (7.3%) | 4 (5.3%) | 5 (10.4%) | p=0.806  *χ*^2^=1.617 |
| $40 000 - $69 999 | 14 (11.4%) | 8 (10.7%) | 6 (12.5%) |  |
| $70 000 - $119 999 | 37 (30.1%) | 22 (29.3%) | 15 (31.3%) |  |
| $120 000 - $149 999 | 12 (9.8%) | 8 (10.7%) | 4 (8.3%) |  |
| >$150 000 | 51 (41.5%) | 33 (44.0%) | 18 (37.6%) |  |

Families were able to report multiple ethnicities, and the resulting answer is a combination of both parents indicating if they were any of the ethnicities; as such ethnicity totals do not add to n. Ethnicity proportions are provided out of 303 (total), 121 (used DA) and 182 (did not use DA), representing the number of parents reporting ethnicity.

Families who had only received a partial diagnosis were counted as “no diagnosis”, due to a presumption of continued information seeking.

Main language used, highest level of education, income are all obtained from surveys and thus there is some missing data.

# **Supplementary Table 2 – Pre- and post-screening psychosocial outcomes compared by use of GA**

|  | **Participated in screening** | **Used GA** | **Did not use GA** | **Significance** |
| --- | --- | --- | --- | --- |
| ***Survey 2 (pre-screening)*** | | | |  |
| Decisional conflict score (DCS) (20), n(%) [n=95] – measures uncertainty in decision-making | | | | |
| Low (<25) | 74 (77.9%) | 49 (73.1%) | 25 (89.3%) | p=0.165  *χ*^2^=3.603 |
| Moderate (25-37.5) | 18 (19.0%) | 16 (23.9%) | 2 (7.1%) |  |
| High (>37.5) | 3 (3.2%) | 2 (3.0%) | 1 (3.6%) |  |
| State-Trait Anxiety Inventory (STAI) (21,22), n(%) [n=90] – characterization of current or anticipated stressful state | | | | |
| Low (<31) | 35 (38.9%) | 23 (35.9%) | 12 (46.2%) | p=0.661  *χ*^2^= 0.827 |
| Moderate (31-49) | 40 (44.4%) | 30 (46.8%) | 10 (38.5%) |  |
| High (>49) | 15 (16.7%) | 11 (17.2%) | 4 (15.4%) |  |
| ***Survey 3 (post-screening)*** | | | |  |
| Decision Regret Scale (DRS) (23) [n=48], n(%) – measures distress or remorse after a healthcare decision | | | | |
| No regret (0) | 22 (45.8%) | 18 (45.0%) | 4 (50.0%) | p=0.531  *χ*^2^= 1.266 |
| Low regret (1-25) | 19 (39.6%) | 17 (42.5%) | 2 (25.0%) |  |
| Moderate-strong regret (>25-100) | 7 (14.6%) | 5 (12.5%) | 2 (25.0%) |  |
| Genomics Outcome Scale (GOS) (24), mean (SD) [n=47] – measures understanding, decision-making, and emotional regulation(out of 100) | | | | |
| Score | 68.9 (14.6) | 68.6 (15.3) | 70.3 (11.2) | t=0.3018  p=0.764 |
| Feelings About genomic Testing Results (FACToR) (25) [n=48], mean(SD) – measures psychosocial impact of receiving genomic findings | | | | |
| Privacy concerns^a^ (0-8) | 1.2 (1.7) | 0.9 (1.1) | 2.8 (3.0) | p=0.117 |
| Uncertainty^a^ (0-12) | 1.6 (2.1) | 1.4 (1.8) | 2.8 (3.4) | p=0.489 |
| Positive feelings^a^ (0-16) | 3.8 (2.9) | 3.9 (3.0) | 3.6 (2.4) | p=0.993 |
| Negative emotions^a^ (0-12) | 1.4 (1.3) | 1.3 (1.2) | 2.2 (1.9) | p=0.196 |
| Satisfaction with genetic counseling (26) [n=44], mean (SD) – rating experience across 7 domains | | | | |
| Score (0 - 5)^a^ | 4.8 (0.4) | 4.8 (0.4) | 4.7 (0.5) | p=0.768 |
| ***Answered across multiple surveys*** | | | | |
| Threatening Medical Situations Inventory (TMSI) (27) [n=128], mean(SD) – tendency to seek or avoid information under stress | | | | |
| Cognitive confrontation | 19.5 (4.8) | 20.5 (4.0) | 17.8 (5.5) | **p=0.002** |
| Cognitive avoidance^a^ | 20.1 (4.6) | 19.9 (4.6) | 20.5 (4.6) | p=0.428 |

^a^ non-normally distributed data; non-parametric testing used
